# Suspected Energy Drink–Induced Acute Coronary Syndrome With Sudden Cardiac Arrest

**DOI:** 10.1155/cric/1202116

**Published:** 2025-08-26

**Authors:** Priya Ramcharan, Nicholas Pereira, Matthew Maharaj, Arun Katwaroo, Valmiki Seecheran, Rajeev Seecheran, Dayna Lalchansingh, Naveen Seecheran

**Affiliations:** ^1^Department of Medicine, North Central Regional Health Authority, Mt. Hope, North, Trinidad and Tobago; ^2^Department of Medicine, North West Regional Health Authority, Port of Spain, North, Trinidad and Tobago; ^3^Division of Nephrology, University of New Mexico Medical Center, Albuquerque, New Mexico, USA; ^4^Department of Clinical Medical Sciences, University of the West Indies, St. Augustine, North, Trinidad and Tobago

**Keywords:** acute coronary syndromes (ACSs), arrhythmias, energy drinks, sudden cardiac arrest (SCA), ventricular fibrillation (VF)

## Abstract

Overconsumption of energy drinks containing high levels of caffeine has been increasingly linked to cardiovascular morbidity and mortality. This case report describes a 24-year-old Caribbean–Black male with no prior comorbidities who experienced an aborted sudden cardiac death (SCD) after a recent energy drink binge a few hours prior to his ventricular fibrillation (VF) cardiac arrest. Primary percutaneous coronary intervention (PPCI) was successfully performed for a dreaded widowmaker lesion, thought to have arisen as a sequela of his excessive energy drink intake. The clinician should be cognizant of the major adverse cardiovascular events (MACEs), such as acute coronary syndromes (ACSs) and lethal arrhythmias, implicated with energy drink consumption.

## 1. Introduction

Energy drink consumption has escalated globally, particularly among young adults, driven by aggressive marketing campaigns alluding to enhanced physical and mental performance [1–3]. These beverages often contain high concentrations of caffeine, taurine, and other stimulants, with the former being the primary active ingredient responsible for their stimulant effects [4, 5]. While caffeine is generally regarded as safe in moderate amounts, excessive intake through energy drinks has been linked to major adverse cardiovascular events (MACEs), including hypertension, arrhythmias, and acute coronary syndromes (ACSs) [6–9]. This case report describes a young male with aborted sudden cardiac death (SCD) and ventricular fibrillation (VF) likely precipitated by excessive consumption of an energy drink.

## 2. Case Presentation

A 24-year-old Caribbean–Black male with no significant medical history presented to the emergency department following an episode of new-onset, unstable angina following an energy drink binge (approximately 10 cans within the previous 2–3 h [Monster Energy drink, original, 16 oz, Monster Beverage Corporation, Corona, California, United States]). While being evaluated by the junior emergency medicine resident, he experienced sudden cardiac arrest (SCA) with VF and was successfully resuscitated as per Advanced Cardiac Life Support (ACLS) protocol with return of spontaneous circulation (ROSC) without any significant delay (subsequently after the first defibrillation attempt with 200 J). He did not have any family history of premature coronary artery disease or SCD or pertinent social history of alcohol, tobacco, or illicit drug abuse. He detailed a history of long-term energy drink consumption, averaging five energy drinks per day, which he used to manage fatigue from his academic and recreational sports activities.

Postresuscitation, his vital signs were blood pressure of 176/102 mmHg, tachycardia at 122 beats per minute, tachypnea at 24 breaths per minute with an oxygen saturation of 98% on a fractional inspired oxygen of 60% via a nonrebreather (NRB) mask, and an afebrile temperature. He appeared awake, alert, and oriented without any focal neurological deficit. His cardiopulmonary examination revealed auscultated S_1_ and S_2_, with no additional sounds or murmurs. His jugular venous pulse was normal, and his apical impulse was prominent but undisplaced. There were no bronchial breath sounds or pulmonary crackles. The abdominal examination was soft and nontender, with normal bowel sounds. There was no evidence of sacral or peripheral edema.

**Figure 1:**
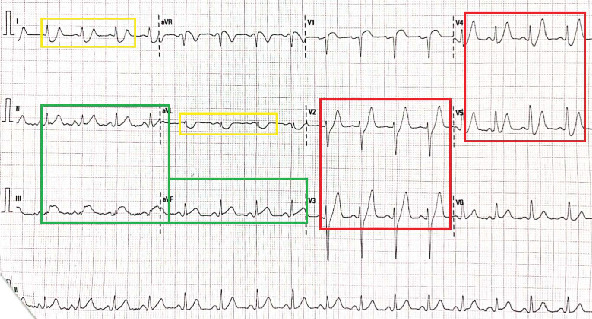
The patient's 12-lead electrocardiogram (ECG) with de Winter T-waves in V_2_–V_5_ (red boxes) and significant ST-segment elevations in II, III, aVF, and V_1_ (green boxes), with reciprocal depressions in I and aVL (yellow boxes).

**Figure 2:**
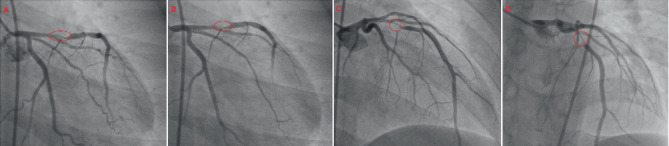
The patient's 95% ruptured plaque in the proximal left anterior descending artery (American College of Cardiology/American Heart Association Type B lesion). (A) A right anterior oblique (RAO)–caudal view illustrating the 95% stenosis within the red ellipse. (B) A right anterior oblique (RAO)–caudal view with a single frame (in red) demonstrating haziness, consistent with Thrombolysis in Myocardial Infarction (TIMI) Thrombus Grade 1. (C) A right anterior oblique (RAO)–cranial view again demonstrating the critical 95% lesion encircled in red. (D) A straight–cranial view with the subocclusive, hazy lesion identified within the red border.

**Figure 3:**
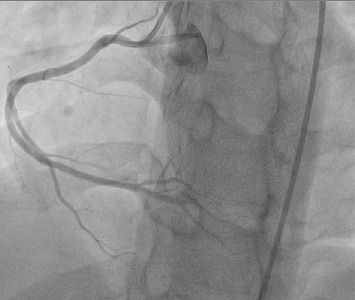
The patient's angiographically normal right coronary artery (RCA).

**Figure 4:**
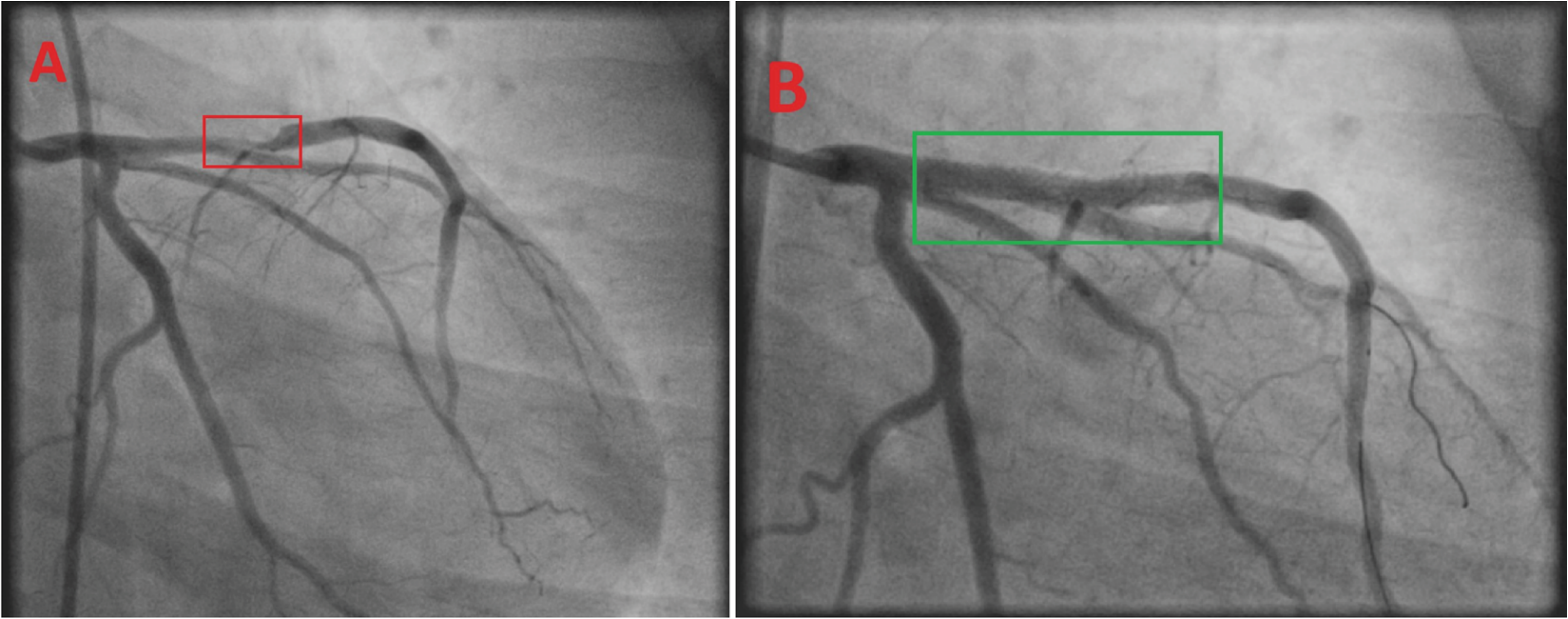
The patient's primary percutaneous coronary intervention (PPCI). (A) The patient's diagnostic coronary angiogram revealed a 95% ruptured plaque in the proximal left anterior descending artery (American College of Cardiology/American Heart Association Type B lesion) (red box). (B) He was subsequently stented with a zotarolimus-eluting stent with an excellent angiographic result and no complications with resultant Thrombolysis in Myocardial Infarction (TIMI) 3 antegrade flow (green box).

His post-ROSC 12-lead electrocardiogram (ECG) revealed sinus tachycardia with de Winter T-waves in V_2_–V_5_ [10] and significant ST-segment elevations in II, III, aVF, and V_1_, with reciprocal depressions in I and aVL (Figure 1). A quick, bedside two-dimensional transthoracic echocardiogram revealed severe hypokinesis of the basal, mid, and apical anterior walls with a reduced left ventricular ejection fraction of 30%–35% and no severe valvular heart disease or pulmonary hypertension. He was immediately initiated on the institutional ACS pharmacotherapy protocol, which comprised aspirin, ticagrelor, and high-intensity rosuvastatin, and proceeded directly for transradial primary percutaneous coronary intervention (PPCI). His preprocedural complete blood count and basic metabolic panel, along with additional electrolyte testing (calcium, magnesium, and phosphorus), were normal, as were the glycated hemoglobin, fasting lipid panel, and thyroid cascade panel. Urine toxicology was also negative for alcohol, cannabis, opiates, and cocaine. His coronary angiography revealed a 95% ruptured plaque in the proximal left anterior descending (LAD) artery (American College of Cardiology/American Heart Association Type B lesion), deemed the culprit for his index ACS and VF cardiac arrest, with an angiographically normal right coronary artery (RCA) (Figures 2 and 3). He was subsequently stented with a zotarolimus-eluting stent with excellent angiographic result and no complications, with resultant Thrombolysis in Myocardial Infarction (TIMI) 3 antegrade flow (Figure 4).

He was subsequently admitted to the cardiac care unit and was commenced on comprehensive guideline-directed heart failure therapy with enalapril, carvedilol, finerenone, and empagliflozin. During the ensuing hospitalization, his clinical status stabilized as he did not report any alarming cardiovascular symptoms or display any features of heart failure or life-threatening arrhythmias. His preliminary workup for autoimmune conditions and thrombophilia returned negative. He was discharged with dual antiplatelet therapy for his recent PCI, a high-intensity statin for his ACS, along with four-pillar HF therapy. He was extensively counseled with respect to lifestyle modification, medication adherence, and energy drink cessation. A 3-month outpatient follow-up revealed interval normalization of his left ventricular ejection fraction, with no serious clinical adverse events, and self-reported continued cessation of energy drinks.

## 3. Discussion

Energy drinks are increasingly consumed worldwide, especially among young adults, for their purported ability to enhance performance and reduce fatigue [11]. Almost one-third of this subpopulation consumes energy drinks regularly, with the vast majority exceeding the recommended daily caffeine intake of 400 mg, largely attributed to aggressive marketing campaigns [12]. A typical 16-oz (473 mL) can of energy drink contains approximately 160 mg of caffeine, which equates to the caffeine content in about four 8-oz cups of black tea or two 8-oz cups of brewed coffee [13]. Additionally, they often contain a high admixture of sugar (up to 54 g per can), taurine, guarana, and B vitamins, all of which contribute to their stimulant and metabolic effects [8, 14]. In this specific case, the Monster Energy drink also contained carbonated water, sucrose, glucose, citric acid, natural flavors, taurine, sodium citrate, color added, panax ginseng root extract, L-carnitine L-tartrate, caffeine, sorbic acid, benzoic acid, niacinamide, sodium chloride, glycine max glucuronolactone, inositol, guarana seed extract, pyridoxine hydrochloride, sucralose, riboflavin, maltodextrin, and cyanocobalamin. Generally, these beverages retail for approximately $2–$4 per 16-oz can, making them a relatively affordable option [15, 16]. The combination of these ingredients has raised concerns about their safety profile, and a growing body of evidence demonstrates a dose–response relationship between energy drink consumption and MACE, particularly in young adults who regularly engage in high-intensity physical activity during active consumption [17–19].

The cardiovascular effects of excessive energy drink consumption are primarily mediated by caffeine, which functions as an adenosine receptor antagonist [6]. This antagonism results in accentuated sympathetic nervous system activation, characterized by increased endogenous catecholamine release with resultant tachycardia and augmented myocardial contractility [6]. This complex milieu creates a perfusion-demand mismatch, predisposing individuals to ischemia, which is critical in triggering MACEs [6]. Chronic consumption has also been inextricably linked to endothelial dysfunction and platelet hyperreactivity [20–22]. Impaired nitric oxide bioavailability coupled with heightened oxidative stress is a key contributory mechanism of microvascular injury, which can potentiate vascular stiffness and atherogenesis [23]. In this case, the patient's acute consumption of these energy drinks, which may have been chronic, may have contributed to the ACS, as evidenced by the ruptured plaque and hazy thrombus resulting from the pathophysiological processes described above.

Additionally, caffeine increases cardiomyocyte sarcoplasmic calcium levels through the phosphodiesterase-cyclic adenosine monophosphate (cAMP) cascade amplification pathway, which enhances myocardial excitability and leads to arrhythmogenic effects [6, 24, 25]. A study by Basrai et al. [26] demonstrated that acute consumption of energy drinks can lead to QT_c_ prolongation, which is robustly implicated in lethal arrhythmias. These mechanistic effects may have contributed to VF cardiac arrest. Energy drinks also present unique cardiovascular risks apart from caffeine, as they often comprise sugar, taurine, and other stimulants. Taurine, while potentially cardioprotective in moderation, can exhibit increased inotropy, which, along with caffeine-mediated effects, can exacerbate myocardial ischemia [27–29]. Acute hyperglycemia resulting from high sugar content is also linked to worsening insulin resistance, thereby amplifying conventional cardiovascular risk factors [30, 31].

This case is interesting from a few aspects: (1) it illustrates the dual-linked devastating complications of ACSs with a ruptured plaque with a lethal arrhythmia in a previously young, healthy male with no other incipient culprit other than recent excessive consumption of energy drinks. Benjo et al. [32] described a patient with a de novo, severe left main coronary artery lesion associated with energy drink consumption, underscoring the role of stimulants in precipitating thrombotic events in previously healthy individuals. Additionally, Pallangyo [33] described a similar patient to ours with a 100% thrombotic occlusion of the proximal LAD artery after recent intake. We acknowledge that there is no absolute, definitive link between the ACS and another undiagnosed condition, such as a hypertensive crisis or thrombophilia (not exhaustively ruled out); however, the signal between the two events—recent intake and aborted SCD—cannot simply be overlooked. This lesion could be attributed to either severe coronary vasospasm or spontaneous coronary artery dissection; however, we lacked the intravascular ultrasound and optical coherence tomography capabilities to ascertain the pathological mechanism definitively. Additionally, the lesion displayed haziness, consistent with a TIMI Thrombus Grade 1, which supported our rationale for a new-onset ruptured plaque.

Secondly, there is a paucity of case reports similarly demonstrating this life-threatening presentation; thus, further large-scale studies are required to establish a causal epidemiologic link and investigate genetic, phenotypic, and ethnic susceptibility to caffeine-related adverse events, as many of the reported cases occurred in patients of black ancestry [34–36]. Energy drinks can potentially trigger latent, pre-existing genetic conditions into lethal arrhythmias and SCD [37]. For example, they exert significant hypertensive effects in patients with long QT syndrome (LQTS). Additionally, 5% of aborted SCA cases were closely associated with recent consumption of an energy drink [38]. Furthermore, large-scale studies are needed to determine this precise risk, especially since these beverages remain unregulated by the Food and Drug Administration (FDA) [39, 40]. Finally, regulatory measures such as outlining safe caffeine consumption limits, especially for vulnerable subgroups, and mandating warning labels are essential for guiding public health policies in this emerging public health hazard [1, 41].

## 4. Conclusion

This case underscores the potentially lethal risks associated with excessive energy drink consumption, particularly in young adults without traditional risk factors for cardiovascular disease. Emergent resuscitation, followed by PPCI with heart failure therapy and energy drink consumption cessation, was critical in attenuating morbidity and mortality. Increased awareness among healthcare providers, patients, and policymakers is essential to mitigate the growing burden of energy drink–related adverse outcomes.

## Data Availability

All available data can be obtained by contacting the corresponding author.
